# Association between physical activity and resistant hypertension in treated hypertension patients: analysis of the national health and nutrition examination survey

**DOI:** 10.1186/s12872-023-03303-x

**Published:** 2023-06-07

**Authors:** Weidai Zhang, Ronghe Xu, Zhixiong Cai, Xiaodong Zheng, Meiyi Zheng, Chumin Ni

**Affiliations:** Department of Cardiology, Shantou Central Hospital, No. 114 Waima Road Shantou, Guangdong, 515000 China

**Keywords:** Physical activity, Resistant hypertension, National Health and Nutrition Examination Survey, Global physical activity questionnaire, Prevalence, Risk

## Abstract

**Introduction:**

Current guidelines suggest that regular aerobic training might lower blood pressure in hypertensive individuals. However, evidence linking resistant hypertension (RH) with total daily physical activity (PA), including work-, transport-, and recreation-related PA, is limited. Therefore, this study assessed the association between daily PA and RH.

**Method:**

A cross-sectional study was conducted using data acquired from a nationwide survey in the US (the National Health and Nutrition Examination Survey, NHANES). The weighted prevalence of RH was calculated, and moderate and vigorous daily PA was assessed using the Global Physical Activity Questionnaire (GPAQ). A multivariate logistic regression model determined the association between daily PA and RH.

**Results:**

A total of 8,496 treated hypertension patients were identified, including 959 RH cases. The unweighted prevalence of RH among treated hypertension cases was 11.28%, while the weighted prevalence was 9.81%. Participants with RH had a low rate of recommended PA levels (39.83%), and daily PA and RH were significantly associated. PA exhibited significant dose-dependent trends with a low probability of RH (p-trends < 0.05). Additionally, participants with sufficient daily PA had a 14% lower probability of RH than those with insufficient PA [fully adjusted odds ratio (OR) = 0.86; 95% confidence interval (CI) = 0.74–0.99).

**Conclusion:**

The present study revealed that RH has an incidence of up to 9.81% in treated hypertension patients. Hypertensive patients tended to be physically inactive, and insufficient PA and RH were significantly associated. Sufficient daily PA should be recommended to reduce the RH probability among treated hypertension patients.

## Introduction

Uncontrolled hypertension is a critical cardiovascular risk factor that increases the risk of coronary artery disease, heart failure, stroke, and chronic kidney disease progression [1]. However, many hypertensive patients develop resistant hypertension (RH) after treatment. RH is defined as a condition in which the blood pressure remains above the recommended levels despite administering optimum doses of three or more classes of antihypertensive drugs, including a diuretic, or if target blood pressure control requires four or more classes of antihypertensive drugs [2–6]. Previous studies have revealed that RH is strongly associated with a high risk of cardiovascular events and all-cause mortality [1, 7].

Besides medication, current guidelines recommend lifestyle modification for blood pressure control in RH patients, including weight reduction, consuming a diet rich in fruits and vegetables, low-fat dairy products, a low-salt diet, smoking cessation, and regular aerobic physical activity (PA) [4, 6, 8]. Regular PA can reduce the blood pressure of individuals with hypertension [9]. A Consensus Document from the European Association of Preventive Cardiology (EAPC) and the ESC Council on Hypertension recommends regular aerobic training in hypertensive patients, while dynamic resistance training is recommended in high-normal hypertension patients [10]. However, evidence on PA implementation for blood pressure management has been relatively limited in prescribed exercise [9]. Additionally, the association between RH and the total amount of daily PA, including work-, transport-, and recreation-related PA, remains unclear. Therefore, we determined the association between daily PA and RH using data from a nationwide US survey (the National Health and Nutrition Examination Survey, NHANES). We hypothesized that sufficient daily PA could reduce the RH probability in treated hypertension patients.

## Methods

### Study Population

First, we retrieved data from the National Health and Nutrition Examination Survey (NHANES) conducted by the National Center for Health Statistics. The NHANES uses data collected from a cross-sectional sample of the noninstitutionalized US civilian population to evaluate its health and nutritional status [11]. About 12,000 people are required to participate in the NHANES every two-year cycle. The percentage agreeing to participate varies from year to year. Of the initial 12,000, an average of 10,500 agreed to complete home interviews, and about 10,000 finished the data collection in the Mobile Test Center [12]. All procedures of the NHANES were approved by the National Center for Health Statistics institutional review board and written informed consent was obtained from all participants.

**Fig. 1:**
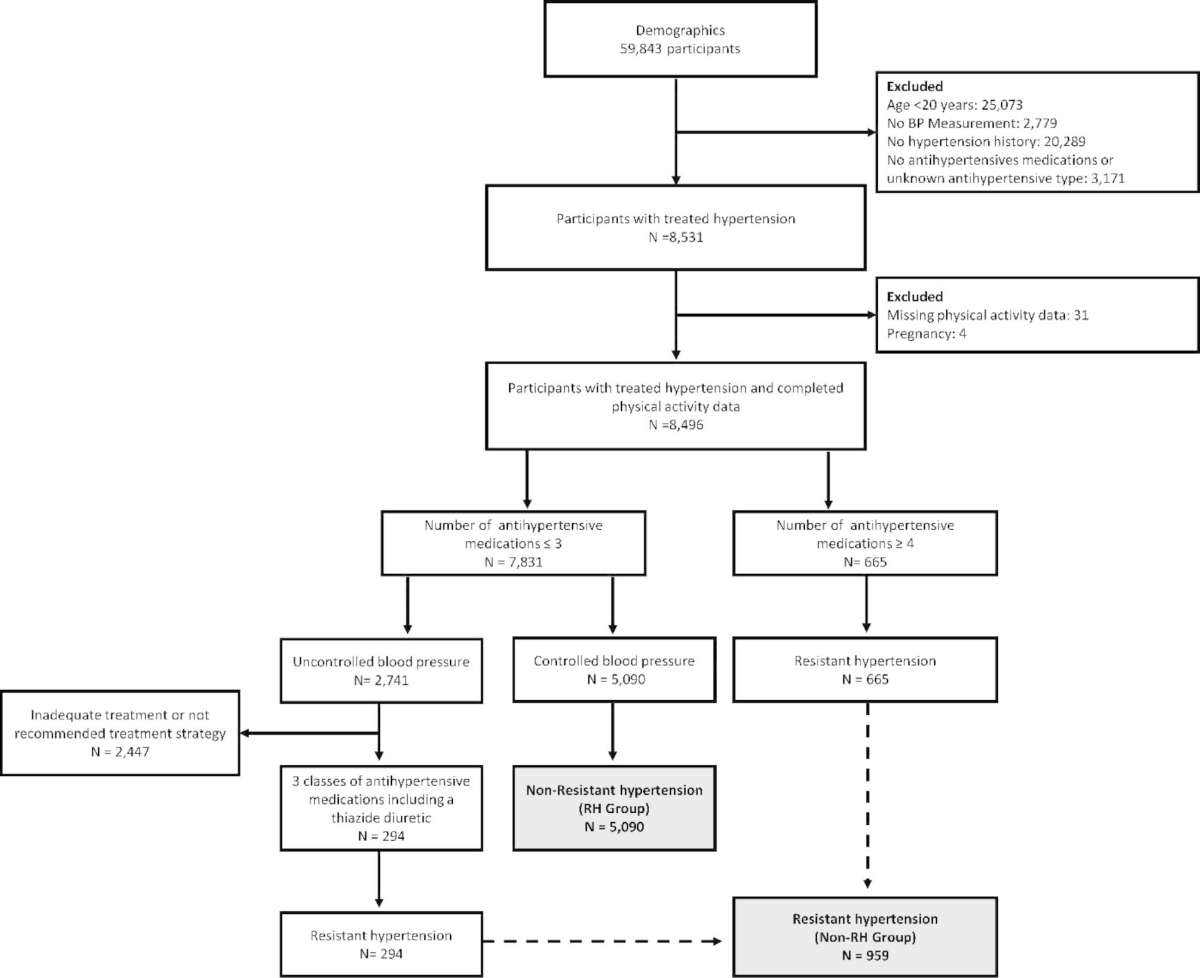
Flowchart illustrating the recruitment criteria of the study population

We examined data from seven consecutive NHANES cycles (2005–2018). We included nonpregnant participants > 20 years old, comprising 45,000 participants. Then, we excluded those with missing blood pressure and PA questionnaire data. Next, we excluded participants under 20 years, pregnant, had no history of hypertension, had not used antihypertensive medication, and had not received adequate antihypertensive therapy (defined as blood pressure ≥ 140/90 mmHg but received less than three antihypertensive medications, or had not used diuretics) (Fig. 1). The final study population consisted of 5000 participants.

### Definition of terms

The NHANES complied with current guidelines for blood pressure measurement [13, 14]. Three consecutive blood pressure measurements were taken to obtain an accurate measurement, and the average values of three consecutive systolic blood pressure (SBP) and diastolic blood pressure (DBP) measurements were calculated. The nurses who took blood pressure measurements obtained certification through a Shared Care Research and Education Consulting training program. Resistant hypertension (RH) was defined as a condition in which the blood pressure remains above the recommended levels despite the administration of optimum doses of three or more classes of antihypertensive drugs, including a diuretic, or if target blood pressure control requires four or more classes of antihypertensive drugs [2–6]. Uncontrolled blood pressure was defined as an average SBP > 140 mmHg or DBP > 90 mmHg while on treatment. Antihypertensive medication use was self-reported.

### Assessment of physical activity

Participants were asked to complete the Global Physical Activity Questionnaire (GPAQ) to assess PA [15, 16]. The GPAQ was developed by the World Health Organization (WHO) in 2002 in response to a greater interest in the role of PA in health [15, 16]. It collects information based on three domains (work, travel to and from places, and recreational activities) and sedentary behavior.

The WHO recommends that adults perform moderate-intensity PA for at least 150 min, vigorous-intensity PA for 75 min, or an equivalent combination of moderate- and vigorous-intensity PA achieving at least 600 metabolic equivalents (MET) minutes every week (Table 1) [16]. Similarly, according to the Physical Activity Guidelines for Americans, adults must engage in moderate-intensity PA for at least 150–300 min, vigorous-intensity aerobic PA for 75–150 min, or an equivalent combination of moderate- and vigorous-intensity aerobic PA every week (Table 1) [17]. In the present study, if the exercise intensity and time met the above requirements, it was considered PA sufficient. Otherwise, it was defined as PA insufficient. Additionally, the MET-minutes per week was calculated to estimate PA and obtain continuous variables for the dose-dependent analysis. Moreover, American guidelines suggest that doing PA beyond the equivalent of 300 min of moderate-intensity PA can provide additional health benefits. Therefore, participants who surpassed this were described as having overfulfilled PA (PA overfulfilled). A detailed description is presented in Table 1.

**Table 1:** Recommended physical activity levels per week

		Recommendation A ^a^	Recommendation B ^a^	Recommendation C ^a^	Recommendation D ^a^
Recommendations by WHO [16]	Time spent	At least 150 min of moderate-intensity PA	At least 75 min of vigorous-intensity PA		
	MET values ^b^	At least 150 min * 4.0 **= 600 MET-minutes** ^c^	At least 75 min * 8.0 **= 600 MET-minutes** ^d^	Equivalent combination of moderate- and vigorous-intensity PA **> 600 MET-minutes** ^e^	
Guideline for Americans [17]	Time spent	150 to 300 min of moderate-intensity PA	75 to 150 min of vigorous-intensity PA	Equivalent combination of moderate- and vigorous-intensity PA	Beyond the equivalent of 300 min of moderate-intensity PA

PA, physical activity; METs, metabolic equivalents; WHO, World Health Organization

^a^ Based on the recommendations of the WHO and the American guidelines, participants who meet either Recommendation A, Recommendation B, or Recommendation C are described as having achieved sufficient physical activity (PA_sufficient_). Participants who meet Recommendations D are described as having overfulfilled physical activity (PA_overfulfilled_).

^b^ MET values are applied to the time variables to assess the intensity (moderate or vigorous) of PA.

^c^ MET values of moderate-intensity work, moderate-intensity recreation, and transport (cycling and walking) are equivalent to 4.0

^d^ MET values of vigorous-intensity work and vigorous-intensity recreation are equivalent to 8.0

^e^ An equivalent combination of MET values of moderate- and vigorous-intensity physical activity

### Covariates

We included demographic variables, smoking status (either ever smoked or have never smoked), body mass index (BMI, kg/m^2^), total cholesterol (mg/dL), aspartate aminotransferase (AST, IU/L), alanine aminotransferase (ALT, IU/L), estimated glomerular filtration rate (eGFR, estimated using the Modification of Diet in Renal Disease 4-variable Eq. 1 [18]), total nutrient intake per day [sodium (mg), energy (kcal), and fat (gm)] as covariates. Demographic variables included sex, age, race (non-Hispanic blacks or other races), family poverty index ratio (PIR), and marital status (married, unmarried, or another status).

### Statistical analysis

Continuous variables are expressed as means ± standard deviations or medians (interquartile ranges, IQR), whereas categorical variables are presented as cases (n) and percentages (%). Non-normal distributions were transformed by conducting natural log or Box-Cox transformations. For comparisons between continuous variables, normally distributed data were analyzed using Student’s *t*-test, whereas non-normally distributed data were analyzed using Wilcoxon signed-rank test. Categorical variables were analyzed using χ [2] tests. To ensure a national representation, the weighted RH prevalence was calculated in line with the analytical guidelines of the National Center for Health Statistics. An unweighted logistic regression model was also applied to analyze the association between PA and RH since cases with special health concerns were excluded. The effect estimates were reported as odds ratios (ORs) and 95% confidence intervals (CIs). Statistical analyses were performed using IBM SPSS Statistics (v. 24.0, IBM Corp., Armonk, NY, USA) and R (v. 3.6.0). A *p* < 0.05 was considered statistically significant.

## Results

### Characteristics of the participants

First, we retrieved data on 59,843 participants from seven consecutive National Health and Nutrition Examination Survey (NHANES) cycles (2005–2018). Among them, 8,496 cases of treated hypertension were screened, including 959 cases of resistant hypertension (RH). The unweighted prevalence of RH among treated hypertension cases was 11.28%, and the weighted prevalence was 9.81%.

After the screening, 6,049 participants were finally enrolled (Fig. 1), including 2852 men and 3197 women aged 20 to 80 years (median age, 63 years). RH participants were older, with a higher proportion of men (50.78% vs. 46.46%) than non-RH. Most participants with hypertension did not meet the recommended daily PA levels (only 46.60% attained the recommended PA levels). The two groups significantly differed for race, family PIR, marital status, BMI, total cholesterol, eGFR, nutrient intake, and ALT (p < 0.05). Detailed descriptions of participant characteristics are presented in Table 2.

**Table 2:** Baseline characteristics of the study population

Characteristics	All patients	Resistant hypertension group N = 959	Non-resistant hypertension group N = 5,090	*P*-value
Age				< 0.001
20–35 years	128 (2.1%)	9 (0.94%)	119 (2.34%)	
36–50 years	869 (14.4%)	80 (8.34%)	789 (15.50%)	
51–65 years	2301 (38.0%)	299 (31.18%)	2002 (39.33%)	
Over 65 years	2751 (45.5%)	571 (59.54%)	2180 (42.83%)	
Sex				< 0.05
Male	2852 (47.1%)	487 (50.78%)	2365 (46.46%)	
Female	3197 (52.9%)	472 (49.22%)	2725 (53.54%)	
Race^1^				< 0.001
Non-Hispanic Blacks	1676 (27.7%)	379 (39.52%)	1297 (25.48%)	
Other races	4373 (72.3%)	580 (60.48%)	3793 (74.52%)	
Family PIR^2^				< 0.01
≤ 1.85	2406 (39.8%)	407 (42.44%)	1999 (39.27%)	
> 1.85	3082 (51.0%)	446 (46.51%)	2636 (51.79%)	
Unknown	561 (9.3%)	106 (11.05%)	455 (8.94%)	
Marital status				< 0.05
Married	3318 (54.9%)	486 (50.68%)	2832 (55.64%)	
Unmarried	2214 (36.6%)	385 (40.15%)	1829 (35.93%)	
Other status	517 (8.5%)	88 (9.18%)	429 (8.43%)	
Former and current smoker	3030 (50.09%)	519 (54.12%)	2511 (49.33%)	< 0.01
Body mass index^3^				< 0.01
< 25 kg/m^2^	873 (14.4%)	118 (12.3%)	755 (14.8%)	
25–30 kg/m^2^	1864 (30.8%)	261 (27.2%)	1603 (31.5%)	
> 30 kg/m^2^	3312 (54.8%)	580 (60.5%)	2732 (53.7%)	
Log (ALT)^4^	3.08 ± 0.44	3.04 ± 0.44	3.09 ± 0.44	< 0.001
Log (AST)^5^	3.18 ± 0.34	3.16 ± 0.33	3.18 ± 0.34	> 0.05
Total cholesterol (mg/dL)	4.79 ± 1.06	4.67 ± 1.11	4.82 ± 1.05	< 0.001
eGFR (mL/min/1.73 m^2^)	76.37 ± 23.86	68.18 ± 24.89	77.92 ± 23.34	< 0.001
Sodium intake (mg/d)	3189.55 ± 1588.37	3066.58 ± 1495.92	3212.72 ± 1604.28	< 0.05
Energy intake (kcal/d)	1893.29 ± 828.03	1807.65 ± 783.65	1909.43 ± 835.22	< 0.001
Fat intake (gm/d)	74.24 ± 40.95	70.61 ± 40.09	74.92 ± 41.08	< 0.05
Metabolic Equivalents (METs)	480.00 (0.00-2160.00)	216.00 (0.00-1440.00)	480.00 (0.00-2400.00)	< 0.001
With sufficient PA	2819 (46.60%)	382 (39.83%)	2437 (47.88%)	< 0.001

eGFR, estimated glomerular filtration rate; PA, physical activity; PIR, poverty index ratio; ALT, alanine aminotransferase; AST, aspartate aminotransferase;

^1^Considering the impact of race on resistant hypertension, race was divided into non-Hispanic blacks and other races (Mexican Americans, other Hispanics, non-Hispanic whites, non-Hispanic Asians, and others);

^2^Below poverty level was defined as PIR < 1.85, above poverty level was defined as PIR > 1.85;

^3^Body mass index (BMI) was calculated as the weight in kilograms divided by the square of the height in meters. Overweight was defined as BMI ≥ 25 kg/m^2^ and < 30 kg/m^2^, and obesity was defined as BMI ≥ 30 kg/m^2^;

^4^Log (ALT), natural logarithm of ALT;

^5^Log (AST), natural logarithm of AST;

The results of the multivariate regression analyses are summarized in Table 3. In the unadjusted model, the PA index (MET-minutes per week, Box-Cox transformed) was negatively correlated with RH (OR = 0.95, 95% CI: 0.93–0.96, p < 0.05). After adjusting for confounding factors, the negative correlation between PA and RH remained significant in model 2 (OR = 0.96, 95% CI: 0.94–0.98, p < 0.001) and model 3 (OR = 0.97, 95% CI: 0.95–0.99, p < 0.001). Daily PA exhibited significant dose-dependent trends with a low RH probability (p-trends < 0.05) when PA was set as the categorical variable (quartiles).

**Table 3:** Association between physical activity and resistant hypertension

	Odds ratios (95% confidence interval)		
	Model 1 ^a^	Model 2 ^b^	Model 3 ^c^
MET-minutes ^d^	0.95 (0.93, 0.96)	0.96 (0.94, 0.98)	0.97 (0.95, 0.99)
Quintiles of MET-minutes			
Quintile 1 (< 1)	1.0	1.0	1.0
Quintile 2 (1-120)	0.91 (0.60, 1.37)	0.94 (0.61, 1.43)	0.93 (0.60, 1.43)
Quintile 3 (121–880)	0.78 (0.65, 0.94)	0.82 (0.68, 1.00)	0.86 (0.71, 1.05)
Quintile 4 (881–2940)	0.72 (0.59, 0.87)	0.78 (0.64, 0.95)	0.85 (0.70, 1.04)
Quintile 5 (≥ 2941)	0.58 (0.47, 0.71)	0.67 (0.54, 0.83)	0.74 (0.60, 0.92)
*P* for trend	< 0.01	< 0.01	< 0.05

^a^ Model 1 was unadjusted

^b^ Model 2 was adjusted for age, sex, race, family poverty index ratio (PIR), and marital status

^c^ Model 3 was adjusted for variables in Model 2 + smoking status, BMI, total cholesterol, aspartate aminotransferase (natural log transformed), alanine aminotransferase (natural log transformed), estimated glomerular filtration rate, dietary sodium, dietary energy, and dietary fat

^d^ Data were Box-Cox transformed. MET-minutes per week were used to assess the intensity (moderate or vigorous) of physical activity

According to the recommendations of the WHO and Physical Activity Guidelines for Americans, patients were classified as PA_sufficient_ and PA_insufficient_ (Table 1). The univariate analysis showed that PA_sufficient_ was significantly associated with RH (*OR* = 0.72; 95% *CI* = 0.63–0.83). After adjusting for demographic variables (model 2), PA_sufficient_ was still significantly associated with RH (*OR* = 0.79; 95% *CI* = 0.68–0.92). In model 3, participants with sufficient PA had a 14% lower probability of RH than those with insufficient PA after adjusting for confounding factors (*OR* = 0.86; 95% *CI* = 0.74–0.99) (Table 4).

**Table 4:** Association between sufficient physical activity and resistant hypertension

	Model A [1]		Model B [2]		Model C [3]	
	*OR P*		*OR P*		*OR P*	
Insufficient physical activity	Reference		Reference		Reference	
Sufficient physical activity	0.72 (0.63,0.83)	< 0.01	0.79 (0.68,0.92)	< 0.05	0.86(0.74,0.99)	< 0.05

RH, resistant hypertension; OR, odds ratio

^[[1]]^ Model A was unadjusted

^[[2]]^ Model B was adjusted for age, sex, race, family poverty index ratio (PIR), and marital status

^[[3]]^ Model C was adjusted for variables in Model 2 + smoking status, BMI, total cholesterol, aspartate aminotransferase, alanine aminotransferase, estimated glomerular filtration rate, dietary sodium, dietary energy, and dietary fat

Moreover, PA_overfulfilled_ was used as a variable for regression analysis based on the Physical Activity Guidelines for Americans. The associations between PA_overfulfilled_ and RH in multivariate sequential models are presented in Table 5. PA_overfulfilled_ participants had a significantly lower probability of RH. The fully adjusted *OR* between overfulfilled and insufficient PA groups was 0.83 (95% *CI* = 0.70–0.98).

**Table 5:** Association between overfulfilled physical activity and resistant hypertension

	Model 1 ^a^		Model 2 ^b^		Model 3 ^c^	
	*OR P*		*OR P*		*OR P*	
**Comparison with insufficient PA group**						
Overfulfilled physical activity group	0.69 (0.59,0.81)	< 0.01	0.77 (0.65, 0.90)	< 0.01	0.83(0.70, 0.98)	< 0.05

OR, odds ratio

^a^ Model 1 was unadjusted

^b^ Model 2 was adjusted for age, sex, race, family poverty index ratio (PIR), and marital status

^c^ Model 3 was adjusted for variables in Model 2 + smoking status, BMI, total cholesterol, aspartate aminotransferase (natural log transformed), alanine aminotransferase (natural log transformed), estimated glomerular filtration rate, dietary sodium, dietary energy, and dietary fat

## Discussion

In the current study, the prevalence of resistant hypertension (RH) was up to 9.81%, slightly lower than previous studies, ranging from 12 to 14% ^2^. This variation could be attributed to the relatively strict screening criteria, excluding treated hypertension patients who received inadequate treatment or had no recommended treatment strategy.

Patients with RH have poor cardiovascular outcomes. Therefore, it is necessary to use a comprehensive treatment strategy to enhance blood pressure control in these patients [1]. Previous studies have presented biological evidence to support the role of PA in lowering blood pressure. Multiple mechanisms are involved in the adaptation of blood pressure to PA [19]. Sufficient PA can reduce systemic vascular resistance via decreased activities of the sympathetic nervous and the renin-angiotensin systems [20]. Moreover, PA can improve endothelium-dependent vasodilatation and enhance baroreceptor sensitivity and arterial compliance [20].

Guidelines recommend lifestyle interventions for managing hypertension [10, 21]. Randomized controlled trials have demonstrated the effects of aerobic PA in blood pressure control [9]. However, evidence for the implementation of PA in blood pressure management has been relatively limited in prescribed exercise. In most previous studies, aerobic activity was defined in detail regarding intensity, frequency and duration. Further, the medical ergometer cycle is the most frequently used way to implement aerobic activities. Notably, many individuals spontaneously participate in daily PA but not in a prescribed scheme [15, 22]. Nevertheless, few studies have investigated the effects of daily PA on hypertension, especially RH. It is unclear whether daily PA, not prescribed exercise, can also contribute to controlling blood pressure. Hence, we determined the relationship between daily PA and RH probability in treated hypertension patients.

Hypertensive patients tended to be physically inactive (46.6%). RH participants had lower rates of meeting the recommended PA levels than those without RH (39.83 and 47.88%, respectively). Previous studies have found that physical inactivity might account for 5–13% of hypertension risk [9]. The current study also observed strong associations between daily PA and RH probability in treated hypertension patients. After adjusting for demographic factors, dietary factors, and laboratory results, sufficient daily PA revealed an important role in lowering RH probability, with clinically relevant treatment effects. Moderate-intensity daily PA for at least 150 min, vigorous-intensity daily PA for at least 75 min, or an equivalent combination of moderate- and vigorous-intensity daily PA effectively reduced RH probability in treated hypertension patients.

Based on our results, the daily PA mentioned above can be recommended to reduce RH probability in treated hypertension patients. Daily PA is as effective as prescribed exercise to reduce RH probability. The present study defines daily PA in three settings: work, travel to and from places, and recreational activities. Specifically, the following moderate-intensity daily PA can be recommended to reduce RH risk [16]: (1) daily work leading to small increases in breathing or heart rate (e.g., brisk walking or carrying light loads), (2) walking or bicycle for commuting, (3) sports, fitness or recreational activities leading to a mildly accelerated breathing or heart rate (e.g., brisk walking, cycling, swimming, volleyball). Moreover, the following vigorous-intensity daily PA can also be recommended [16]: (1) daily work leading to large increases in breathing or heart rate (e.g., carrying heavy loads, digging, or construction work), (2) sports, fitness, or recreational activities leading to significantly accelerated breathing or heart rates (e.g., running or football).

To determine whether there was an additional benefit from further increases in PA, we introduced another variable, the overfulfilled PA, defined as PA beyond 300 min of moderate-intensity PA. Overfulfilled PA could reduce the RH probability by 17% compared to insufficient PA. As mentioned above, moderate-intensity daily PA for at least 150 min or vigorous-intensity daily PA for at least 75 min comprises sufficient PA, reducing the RH probability by 14% compared to insufficient PA. Thus, the advantage of 300 min of moderate-intensity PA (overfulfilled PA) was less pronounced compared to sufficient PA. The effectiveness of the daily PA is probably dose-dependent and increases with increasing dosage. After fully adjusting for confounding factors, daily PA exhibited significant dose-dependent trends with a low RH probability when PA was the categorical variable (quartiles). Based on our results, adverse blood pressure response was not observed in overfulfilled exercising. Consequently, taking as much daily PA as possible could have further increased the reduction in RH probability. However, the additional benefit of overfulfilled PA compared to sufficient PA was small, and adherence to overfulfilled PA is a challenge to most patients. It is more reasonable to recommend basic sufficient PA for most hypertensive patients to reduce the RH probability [23].

### Strengths and limitations

The major strengths of the present study included using a large sample size, which increased the power of our findings. The prevalence of RH among treated hypertension cases was reliable since a nationally representative sample of the US population was used. Additionally, the detailed information collected from the NHANES included a wide range of potential confounders, such as dietary intakes and laboratory results. Moreover, since 1999, the NHANES survey has become a continuous program focusing on various health and nutrition measurements. As a continuous program, it has a mature and reliable sampling process and methodological procedures across the surveys. To the best of our knowledge, the present study is the first to investigate the association between daily PA and RH probability based on a nationwide database. Second, The GPAQ is a valid and reliable tool for measuring PA. Although wearable devices improve the objectivity and accuracy of PA measurement, self-reported survey methods still confer the advantage of covering the general population due to their low cost [15]. Assessment by GPAQ can distinguish the true effect of the daily PA, closer to a real-life spontaneous PA situation, which has led to reliable results for the relationship between daily PA and RH.

However, the present study also has some limitations. First, the cross-sectional design made causal inference problematic. Therefore, further studies involving high-quality prospective cohorts should be conducted to validate the present findings. Second, the blood pressure measurements included only office blood pressure but not ambulatory blood pressure measurements. Third, we did not consider the optimal dosage of each medication. However, medication use and dosage represented real-world management preferences, which effectively reflect PA effects in patients treated for hypertension.

## Conclusion

In summary, we showed that RH has an incidence of up to 9.81% in treated hypertension patients. Hypertensive patients tended to be physically inactive, and most of them, especially RH, did not perform sufficient PA. Furthermore, we found a significant association between insufficient daily PA and RH probability. Thus, sufficient PA should be recommended to reduce RH probability. Although this beneficial effect was dose-dependent and overfulfilled PA presented additional protection against RH, those who achieved sufficient PA (based on the PA guidelines) were also less prone to RH than those insufficiently active.

## Data Availability

Raw data supporting the obtained results are available at the corresponding author.

## Abbreviations

ALT: alanine aminotransferase
AST: aspartate aminotransferase
eGFR: estimated glomerular filtration rate
GPAQ: Global Physical Activity Questionnaire
NHANES: National Health and Nutrition Examination Survey
PA: physical activity
PIR: Poverty index ratio
RH: resistant hypertension
WHO: World Health Organization
